# Deep Learning Based Automatic Left Ventricle Segmentation from the Transgastric Short-Axis View on Transesophageal Echocardiography: A Feasibility Study

**DOI:** 10.3390/diagnostics14151655

**Published:** 2024-07-31

**Authors:** Yuan Tian, Wenting Qin, Zihang Zhao, Chunrong Wang, Yajie Tian, Yuelun Zhang, Kai He, Yuguan Zhang, Le Shen, Zhuhuang Zhou, Chunhua Yu

**Affiliations:** 1 Department of Anesthesiology, Peking Union Medical College Hospital, Chinese Academy of Medical Sciences and Peking Union Medical College, Beijing 100730, China; tianyuan95@pumch.cn (Y.T.); emancipation258@outlook.com (C.W.); tianyajie@pumch.cn (Y.T.); yuelunzhang@outlook.com (Y.Z.); harveyhekai@sina.com (K.H.); zygpumch@126.com (Y.Z.); shenle@pumch.cn (L.S.); 2 Department of Biomedical Engineering, College of Chemistry and Life Science, Beijing University of Technology, Beijing 100124, China; qinwenting@emails.bjut.edu.cn (W.Q.); zhaozihang@emails.bjut.edu.cn (Z.Z.)

**Keywords:** transesophageal echocardiography, deep learning, left ventricle segmentation, transgastric short-axis view, convolutional neural network

## Abstract

Segmenting the left ventricle from the transgastric short-axis views (TSVs) on transesophageal echocardiography (TEE) is the cornerstone for cardiovascular assessment during perioperative management. Even for seasoned professionals, the procedure remains time-consuming and experience-dependent. The current study aims to evaluate the feasibility of deep learning for automatic segmentation by assessing the validity of different U-Net algorithms. A large dataset containing 1388 TSV acquisitions was retrospectively collected from 451 patients (32% women, average age 53.42 years) who underwent perioperative TEE between July 2015 and October 2023. With image preprocessing and data augmentation, 3336 images were included in the training set, 138 images in the validation set, and 138 images in the test set. Four deep neural networks (U-Net, Attention U-Net, UNet++, and UNeXt) were employed for left ventricle segmentation and compared in terms of the Jaccard similarity coefficient (JSC) and Dice similarity coefficient (DSC) on the test set, as well as the number of network parameters, training time, and inference time. The Attention U-Net and U-Net++ models performed better in terms of JSC (the highest average JSC: 86.02%) and DSC (the highest average DSC: 92.00%), the UNeXt model had the smallest network parameters (1.47 million), and the U-Net model had the least training time (6428.65 s) and inference time for a single image (101.75 ms). The Attention U-Net model outperformed the other three models in challenging cases, including the impaired boundary of left ventricle and the artifact of the papillary muscle. This pioneering exploration demonstrated the feasibility of deep learning for the segmentation of the left ventricle from TSV on TEE, which will facilitate an accelerated and objective alternative of cardiovascular assessment for perioperative management.

## 1. Introduction

More than 300 million operations are performed worldwide annually, according to the most recent survey by the World Health Organization [1]. Transesophageal echocardiography (TEE), a cardiovascular assessment technique using a flexible transesophageal probe, is becoming an integral part of perioperative management across a widening range of operations because TEE has demonstrated efficacy in facilitating decision-making during surgeries [2,3] and hemodynamic management for critically ill patients [4,5]. TEE is more practical than transthoracic echocardiography (TTE) during most surgeries, due to operative approaches and sterile requirements. Additionally, TEE is superior to TTE in enhancing the quality of echocardiography by circumventing the acoustic impediments caused by the ribs and lungs [6].

TEE assessment of the left ventricular function and structure is primarily conducted to answer the relatively common and potentially life-threatening problems encountered perioperatively [7,8]. Compared to the long-axis views of TEE, transgastric short-axis views (TSVs) enable facilitated global and local assessments of left ventricular function from the base to the apex by simply adjusting the probe’s depth. TSVs also provide detailed visualization of the layered anatomy of the left ventricular wall during heartbeats. For these reasons, TSV is commonly performed to assess the structure and function of the left ventricle perioperatively.

Perioperative TEE assessment of left ventricle is a time-consuming and experience-dependent procedure, even for seasoned professionals. With advances in medical artificial intelligence (AI), deep learning algorithms are emerging as a supplementary alternative, providing accelerated and objective perioperative cardiovascular assessments [9,10]. While numerous studies have demonstrated improvements in the utilization of deep learning for left ventricular assessment, the majority of them are applicable to TTE analysis [11,12,13,14]. There also has been some research conducted according to TEE images, but focusing on cardiac long-axis views [15,16,17]. On the other hand, due to the evolution of U-Net and its variants since 2015, the segmentation of medical images based on deep learning has shown significant improvement in computational accuracy, sensitivity, and efficiency [18,19,20,21]. The role of U-Net algorithms has been demonstrated in the image segmentation for ovarian lesions [22], brain tumor, liver lesions, lung nodules [23], and so on. However, the feasibility of applying U-Net algorithms to left ventricular segmentation in TSVs remains poorly understood.

The current study aims to evaluate the feasibility of deep learning for automatic segmentation by assessing the validity of different U-Net algorithms. Initially, a large dataset of TSV images was complied from 451 patients undergoing perioperative TEE. Following image preprocessing and data augmentation, the training set was used to train U-Net algorithms for left ventricle segmentation, with the validation set used for checking overfitting. Finally, the test set was used to evaluate and compare the segmentation performance of U-Net algorithms. 

## 2. Materials and Methods

**Figure 1:**
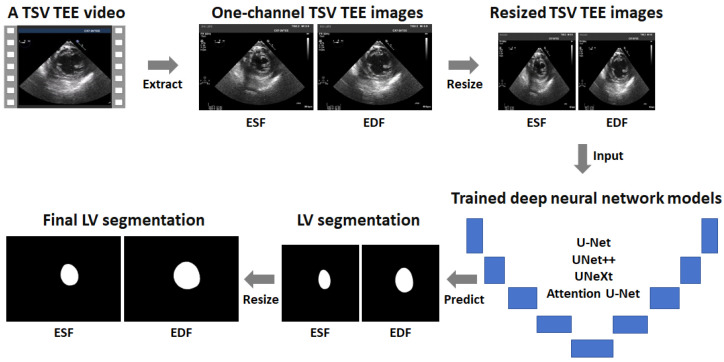
Flow chart of the proposed automatic LV segmentation method for TSV TEE images using deep neural network models. The deep learning models employed were U-Net [18], UNet++ [19], Attention U-Net [20], and UNeXt [21]. LV: left ventricle; TSV: transgastric short-axis view; TEE: transesophageal echocardiography; ESF: end-systolic frame; EDF: end-diastolic frame.

Figure 1 shows the flow chart of the proposed automatic left ventricle segmentation in TSV TEE images using four deep neural network models: U-Net [18], UNet++ [19], Attention U-Net [20], and UNeXt [21]. Firstly, the end-diastolic frame (EDF) and end-systolic frame (ESF) of a TSV TEE video within a cardiac cycle were extracted and converted to one-channel grayscale images. Then, the one-channel ESF and EDF images were resized to a specific size. Subsequently, the resized ESF and EDF images were input to a trained deep neural network model to predict the left ventricle segmentation, which was resized to the original size to obtain the final left ventricle segmentation. The ESF and EDF images were chosen because they were representative and had manual segmentation as the ground truth. This is similar to the EchoNet-Dynamic dataset [11] which also labels only the ESF and EDF images for one TTE video. It should be noted that the trained deep neural network model can be used to segment the left ventricle in any frame of the TSV TEE video. The deep learning networks and the model training will be described in the following subsections.

### 2.1. Patients Enrollment and Dataset Formulation

**Figure 2:**
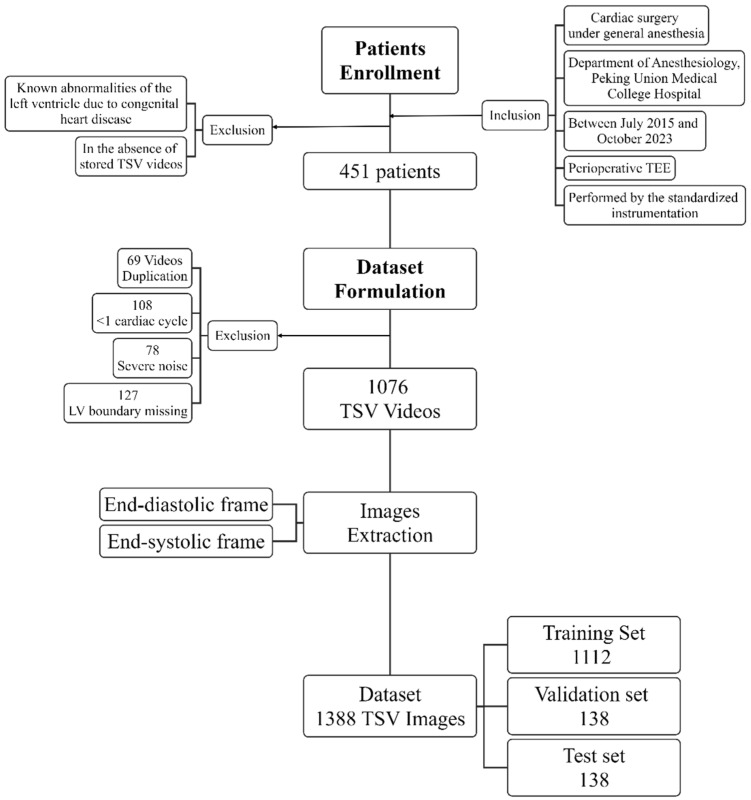
Flow chart of patients enrollment and dataset formulation. TEE: transesophageal echocardiography; TSV: transgastric short-axis view.

This retrospective study was approved by the Ethics Review Committee of the Peking Union Medical College Hospital, Chinese Academy of Medical Sciences. Patients that met the following criteria were involved: (1) those underwent cardiac surgery under general anesthesia at the Department of Anesthesiology, Peking Union Medical College Hospital, between July 2015 and October 2023; (2) those who had perioperative TEE performed by the Philips iE33 ultrasound scanner (Philips *Ultrasound*, Bothell, WA, USA) and the X7-2t transducer (1.0–5.0 MHz). Patients with known abnormalities of the left ventricle due to congenital heart diseases, or without stored videos of TSV were excluded. A total of 1076 TSV videos from the 451 involved patients were enrolled in the current study. Some of the patients had two distinct TSV videos that were acquired pre- and post-cardiac surgery. Of these, as illustrated in Figure 2, 382 videos were considered ineligible because they met any of the following criteria: duplication, coverage of less than one cardiac cycle, significant left ventricle boundary missing, or presence of severe noise. Both the EDF and ESF within a cardiac cycle were extracted from each TSV video, forming the dataset that comprised 1388 images. 

Because a TSV TEE image was a grayscale B-mode ultrasound image, in order to reduce the computational cost of the deep neural network, a 2D three-channel TSV TEE image was converted into a one-channel grayscale image, that is, the number of channels was changed from 3 to 1, so that the amount of computation could be reduced while retaining all the imaging information of the original data. Finally, 1388 2D one-channel TSV TEE images were obtained as the experimental dataset in this study.

**Figure 3:**
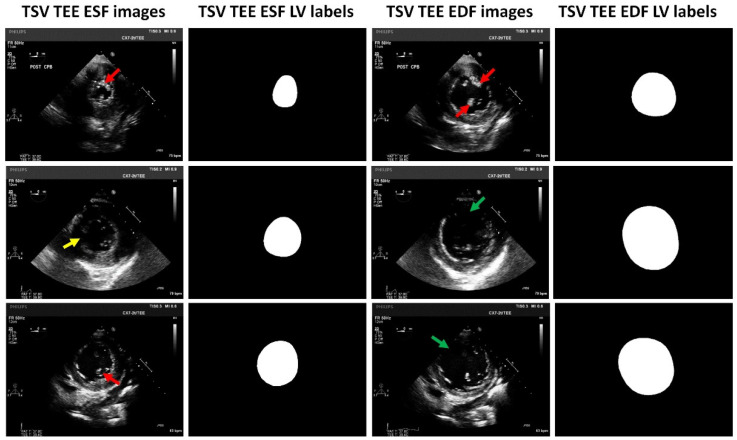
Representative TSV TEE images and corresponding LV labels. Red arrows indicate the papillary muscle. Yellow arrows indicate mild LV boundary missing. Green arrows indicate moderate LV boundary missing. LV: left ventricle; TSV: transgastric short-axis view; TEE: transesophageal echocardiography; ESF: end-systolic frame; EDF: end-diastolic frame.

According to the ratio of 8:1:1, the dataset was randomly divided into a training set, a validation set, and a test set. The training set was used to train the deep neural network models, and the validation set was utilized to check if there was overfitting during the model training, while the test set was employed to evaluate the performance of the trained models. Specifically, the training set contained 1112 TSV TEE images extracted from 556 videos; the validation set contained 138 TSV TEE images extracted from 69 videos; and the test set contained another 138 TSV TEE images extracted from 69 videos. The image data of the same patient did not cross over the training set, the validation set, or the test set to avoid data leakage (i.e., they only appeared in one of the three sets). The manual left ventricle segmentation for each of the 1388 TSV TEE images was performed by two anesthesiologists and confirmed by another senior anesthesiologist, using the open LabelMe software (V3.16.2). The manual segmentation was taken as the ground truth. Figure 3 shows representative TSV TEE images and corresponding manual segmentation as left ventricle labels. As indicated in Figure 3, there are two major challenges for computer-assisted left ventricle segmentation in TSV TEE images: left ventricle boundary missing and papillary muscle interference.

### 2.2. Data Preprocessing and Augmentation

To reduce the computational cost of the deep learning models, each of the 1388 TSV TEE images was subsampled to a size of 256 × 256 pixels using cubic interpolation. Therefore, the size of an input image for the deep neural networks was 256 (image height) × 256 (image width) × 1 (image channel) for both training and testing. Due to the limited amount of experimental data, data augmentation was applied to the images in the training set, including random rotation from 0° to 90°, horizontal flipping, and vertical flipping. Data augmentation can reduce overfitting for the deep neural network model and improve the robustness of the model, which can further improve the generalization ability of the model. Data augmentation was conducted only on the training set, but not on the validation set or the test set. After data augmentation, the size of the training set was increased to 3336. 

### 2.3. Deep Neural Network Models

In this study, four deep neural networks were employed for left ventricle segmentation in TSV TEE images: U-Net [18], UNet++ [19], Attention U-Net [20], and UNeXt [21].

**Figure 4:**
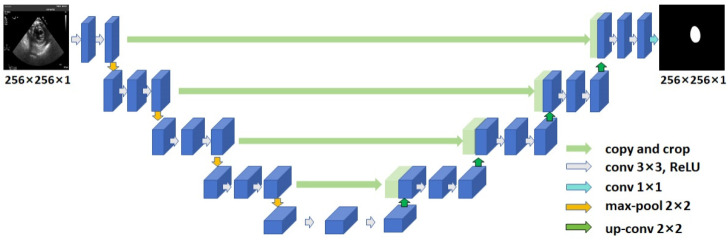
The U-Net network architecture for LV segmentation in TSV TEE images. LV: left ventricle; TSV: transgastric short-axis view; TEE: transesophageal echocardiography; ReLU: rectified linear unit; conv: convolution.

U-Net [18] is the most commonly used and the simplest segmentation model in medical image segmentation, which uses a U-shaped network structure to obtain contextual information and location information (Figure 4). U-Net consists of an encoder and a decoder, with skip connections between the encoder and the decoder. U-Net uses a feature stitching structure to obtain low-level features and high-level semantic features of medical images. The encoding layers of the U-Net network first undergo two convolutional layers to extract features, followed by four down-sampling operations. Similarly, the decoding layers consist of four up-sampling operations and an output module.

**Figure 5:**
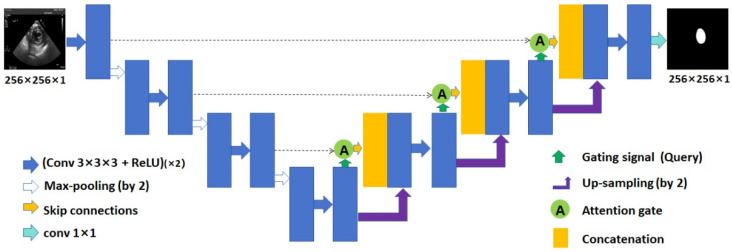
The Attention U-Net network architecture for LV segmentation in TSV TEE images. LV: left ventricle; TSV: transgastric short-axis view; TEE: transesophageal echocardiography; ReLU: rectified linear unit; conv: convolution.

The Attention U-Net [20] model is an extension of the classical U-Net [18] architecture, incorporating the attention mechanism into U-Net (Figure 5), which can gradually strengthen the weight of the local region of interest, suppress the irrelevant regions in the input image, and highlight the salient features of specific local regions.

**Figure 6:**
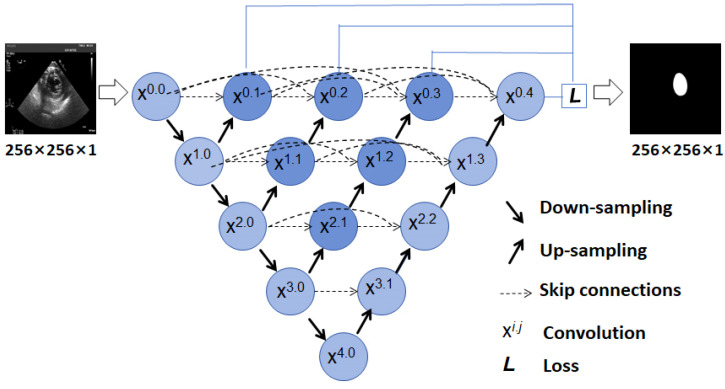
The U-Net++ network architecture for LV segmentation in TSV TEE images. LV: left ventricle; TSV: transgastric short-axis view; TEE: transesophageal echocardiography.

UNet++ [19] is an improvement and extension of the classical U-Net [18] architecture, employing cascaded connections and introducing dense skip connections (Figure 6). It cascades feature maps from both the encoder and decoder, with each decoding layer connected to all deeper encoding layers, forming a dense skip connection structure. This allows the decoder to fully leverage multi-scale features from all encoder layers, making it suitable for scenarios requiring the handling of multi-scale information.

**Figure 7:**
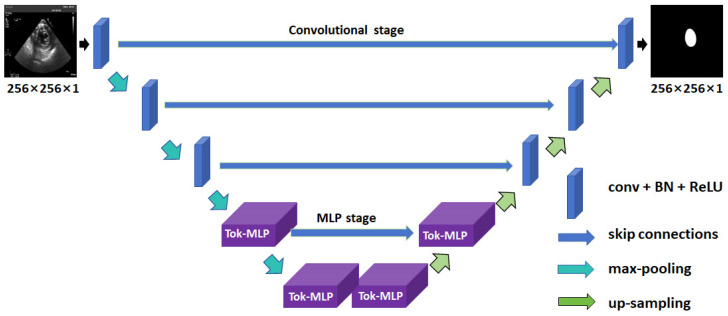
The UNeXt network architecture for LV segmentation in TSV TEE images. LV: left ventricle; TSV: transgastric short-axis view; TEE: transesophageal echocardiography; ReLU: rectified linear unit; BN: batch normalization; conv: convolution; MLP: multi-layer perceptron; Tok: Tokenized.

The UNeXt [21] network’s encoder consists of three convolutional layers and two Tokenized multi-layer perceptron (MLP) modules (Figure 7). In contrast to U-Net [18], UNeXt [21] adopts a leaner approach by employing fewer convolutional layers and larger strides during feature map down-sampling, effectively reducing parameters. UNeXt has gained significant attention as a lightweight solution, emerging as a pioneering fast medical image segmentation network to integrate the MLP module with convolutional layers. At its core lies the Tokenized MLP module, enabling efficient segmentation of medical images with fewer convolutional layers and larger feature down-sampling.

### 2.4. Segmentation Performance Evaluation

In order to evaluate the performance of different deep learning models for left ventricle segmentation in TSV TEE images, the DSC and Jaccard similarity coefficient (JSC) were used as the segmentation performance evaluation metrics:

(1) $$\mathrm{DSC}(A,B)=\frac{2\left|A\cap B\right|}{\left|A\right|+\left|B\right|};$$

(2) $$\mathrm{JSC}(A,B)=\frac{\left|A\cap B\right|}{\left|A\cup B\right|},$$

where *A* represents the left ventricle region predicted by deep learning models, and *B* is the labeled region manually annotated by human experts. Both DSC and JSC ranged from 0 to 1 (or 0% to 100%), with a larger value indicating better segmentation performance.

### 2.5. Experimental Setup

Our experiments were conducted on a graphics workstation with Intel(R) Xeon(R) Gold 6132 CPU@2.60 GHz 2.59 GHz (2 processors), and NVIDIA TITAN RTX 24G, 128G RAM. The PyTorch (version 1.5.1) was used as the deep learning framework. In the experiments, the model input dimensions were 4 (batch size) × 1 (channels) × 256 (height) × 256 (width). The number of training epochs was set at 100. The gradient optimizer was the Adam optimizer. The initial learning rate was set at 10^−3^. The momentum was set at 0.9. A loss function with a combination of the binary cross-entropy (BCE) loss *L*_BCE_ and the DSC loss *L*_DSC_ was used for the U-Net, UNet++, Attention U-Net, and UNeXt models:

(3) $$Loss=\beta L_{\mathrm{BCE}}+\gamma L_{\mathrm{DSC}},$$

where *β* = 0.5, and *γ* = 0.5. *L*_BCE_ and *L*_DSC_ are defined as

(4) $$L_{\mathrm{BCE}}=-B\log(A)-(1-B)\log(1-A),$$

(5) $$L_{\mathrm{DSC}}=1-\frac{2\left|A\cap B\right|}{\left|A\right|+\left|B\right|}$$

where *A* represents the left ventricular region predicted by our model, and *B* is the labeled region manually annotated by human experts. *L*_BCE_ is similar to the cross-entropy loss function, but the binary cross-entropy loss function has an operation to calculate the logit, so we do not need to use the sigmoid function or the softmax function to map the input to [0, 1] for this loss function. According to the official documentation, the binary cross-entropy loss function has better numerical stability than the cross-entropy loss function. *L*_DSC_ is a region-related loss function that has good performance in scenarios where positive and negative samples are seriously unbalanced.

### 2.6. Statistical Analysis

The Kruskal–Wallis test was used to evaluate whether the U-Net, UNet++, Attention U-Net, and UNeXt models had statistically significant differences in terms of the DSC or the JSC for the left ventricle segmentation in the test set of TSV TEE images (*n* = 138). A statistically significant difference was defined as *p* < 0.05. The statistical analysis was performed with IBM SPSS Statistics 27 (IBM Corp., Endicott, NY, USA).

## 3. Results

**Figure 8:**
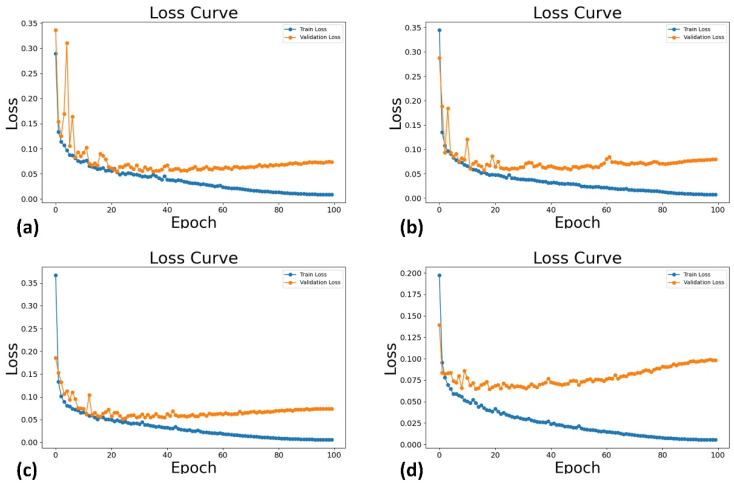
The loss on the training set (i.e., Train Loss) and validation set (i.e., Validation Loss) as a function of training epochs for different deep learning models: Attention U-Net (**a**), U-Net (**b**), UNet++ (**c**), and UNeXt (**d**).

**Figure 9:**
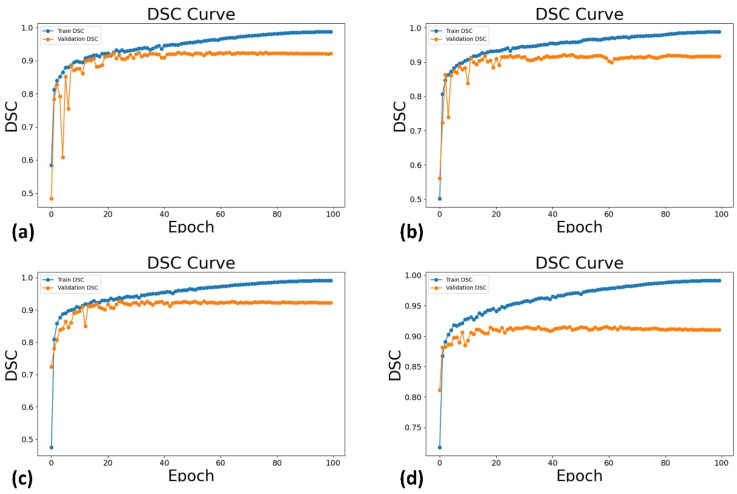
The DSC on the training set (i.e., Train DSC) and validation set (i.e., Validation DSC) as a function of training epochs for different deep learning models: Attention U-Net (**a**), U-Net (**b**), UNet++ (**c**), and UNeXt (**d**). DSC: Dice similarity coefficient.

A total of 1388 images were extracted from 694 TSV videos, featuring 451 patients with an average age of 53.42 years. The analytic population consisted of 32% women, and 27% ASA-PS III or higher. Pre-procedural diagnoses included coronary artery disease, valvular stenosis or regurgitation, aortic disease, and pericardial disease. Figure 8 and Figure 9 show the loss and the DSC on the training set and validation set as a function of training epochs for different deep learning models. For the Attention U-Net, U-Net and UNet++ models, all the training loss and validation loss gradually decreased as the training epochs increased, and they converged when the training epoch reached 100; both the training DSC and validation DSC gradually increased as the training epochs increased, and they converged when the training epoch reached 100. These indicated that the Attention U-Net, U-Net, and UNet++ models had no overfitting or very slight overfitting. The UNeXt model shows similar trends for training loss and DSC, but the validation loss and DSC do not monotonically decrease or increase as the training epochs increased to 100. 

**Figure 10:**
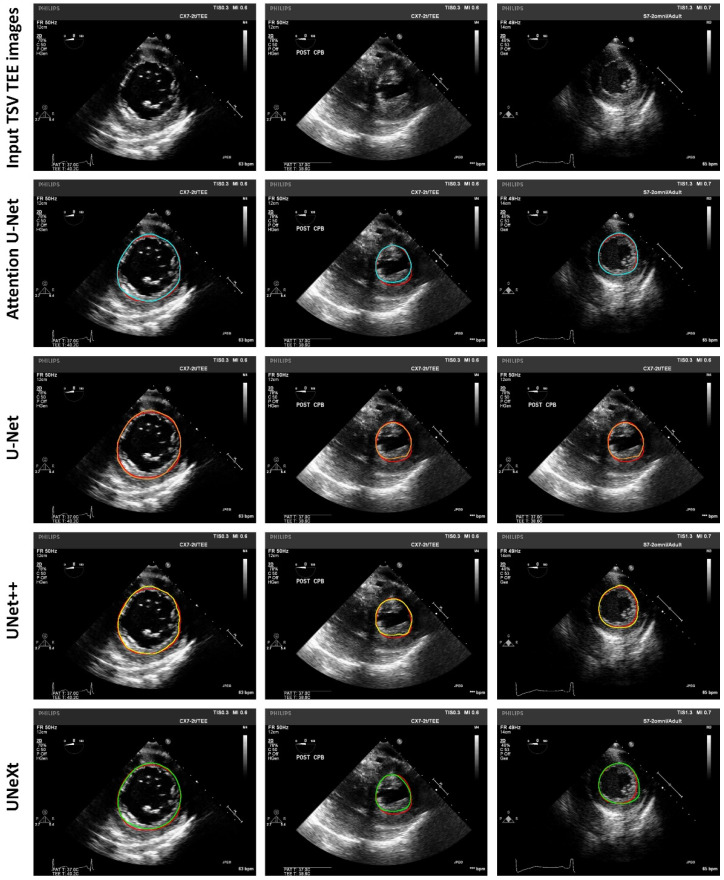
Representative LV segmentation in TSV TEE images using different deep learning models. Red contours indicate manual LV segmentation as the ground truth. Blue contours indicate LV segmentation using Attention U-Net. Orange contours indicate LV segmentation using U-Net. Yellow contours indicate LV segmentation using UNet++. Green contours indicate LV segmentation using UNeXt. TEE: transesophageal echocardiography; TSV: transgastric short-axis view.

Figure 10 shows representative left ventricle segmentation results for TSV TEE images using different deep neural network models. Each column corresponds to a representative TSV TEE case. The first row shows the input images, and the second to the fifth row show the segmented left ventricle contours by the trained Attention U-Net, U-Net, UNet++, and UNeXt models, respectively.

Shown in Table 1 are the U-Net, UNet++, Attention U-Net, and UNeXt models for left ventricle segmentation in TSV TEE images with respect to the number of model parameters, training time (for the 3336 images in the training set), and inference time for a single image. 

The performance comparisons between U-Net and its variants, within the test set (*n* = 138), are shown in Table 2. Kruskal–Wallis test indicated no significant differences in the average JSC and DSC between algorithms.

## 4. Discussion

Very little was found in previous studies on the question of whether U-Net and its variants are feasible for segmentation of the left ventricle from TSV on TEE images. To the best of our knowledge, the current study provides novel evidence of the efficacy and accuracy of deep learning in the expanded medical scenarios.

The encouraging findings indicate that all of the U-Net and its derivatives perform well in the segmentation of the left ventricle from TSV on TEE with an average DSC of 0.91–0.92. These findings are comparable to previous results, which demonstrated ones of 0.92–0.95 for left ventricle segmentation from TTE images [11,12,13,14]. The results also achieve a promising DSC for segmenting the left ventricle from TSV, effectively supplementing the previous studies that used limited TEE images [15,16,17]. From the perspective of using U-Net and its variants, the findings demonstrate superior accuracy in left ventricle segmentation compared to its use in the segmentation of ovarian lesions (0.89) [22], brain tumors (0.89–0.91), liver lesions (0.79–0.83), and lung nodules (0.71–0.77) [23].

Another clinically relevant finding is that the inference times have accelerated to 101–134 ms, compared to the previously reported 230 ms [15]. Given the different tasks focused on in the two studies, a direct comparison between them may not be entirely valid. Nevertheless, the results highlight the proficient performance of U-Net and its variants in segmenting the left ventricle from TSV TEE. It is still noteworthy that the current study uses a CPU as the workstation. It is reasonable to speculate that a GPU workstation would further accelerate the program.

The results of the study indicate no significant differences in accuracy between U-Net and its variants. The absence of significant benefits shown with UNet++ may be due to the standardization of image preprocessing, as it outperforms in enhancing the segmentation quality of various sizes [23]. Compared to U-Net, its variants did not show any improvement. This might be because the simple structure and distinct boundaries of the left ventricle do not require more complex algorithms [14]. The findings demonstrate that slight overfitting existed in the U-NeXt, possibly due to the limited data available in the present study, which may not match the depth of the layers required by the algorithm [20].

In the present study, a large dataset of TEE images was collected, consisting of 1388 images from 451 patients. Restricted by the applicable scenarios, the capacity of this TEE dataset is still incomparable to the international TTE dataset CAMUS, which contains tens of thousands of data points [24]. However, the size of these dataset exceeds the volumes reported in previous studies, which built their deep learning models based on TEE images from 3–95 patients [15,16,17].

**Figure 11:**
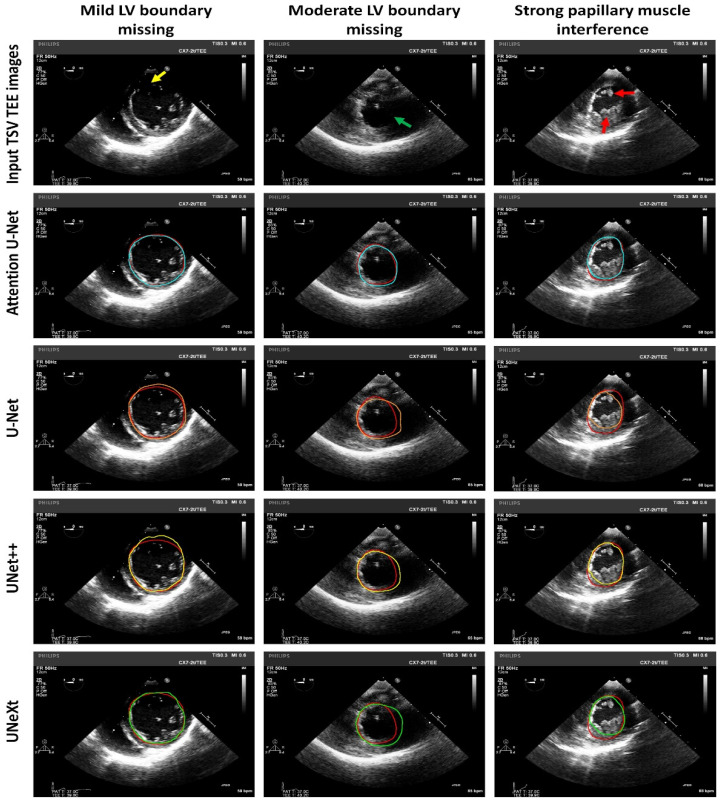
Representative LV segmentation in TSV TEE images for challenging cases of LV boundary missing and strong papillary muscle interference using different deep learning models. Red contours indicate manual LV segmentation as the ground truth. Blue contours indicate LV segmentation using Attention U-Net. Orange contours indicate LV segmentation using U-Net. Yellow contours indicate LV segmentation using UNet++. Green contours indicate LV segmentation using UNeXt. Red arrows indicate the papillary muscle. Yellow arrows indicate mild LV boundaries missing. Green arrows indicate moderate LV boundaries missing. TEE: transesophageal echocardiography; TSV: transgastric short-axis view.

A rather disappointing result is that the current study failed to achieve promising results in left ventricle segmentation for some challenging cases. Figure 11 shows the left ventricle segmentation in representative challenging cases of TSV TEE images. As shown in the first column of Figure 11 which shows a case of mild left ventricle boundary missing, the upper right part of the endocardial boundary is missing in the original image, and the detected boundary in the epicardial region by the U-Net and UNet++ networks is incorrect segmentation; there is slight incorrect segmentation for the UNeXt model. As shown in the second column of Figure 11 which shows a case of moderate left ventricle boundary missing, the left ventricle boundary is missing on the right side in the original image, and there is significant incorrect segmentation for the U-Net, UNet++, and UNeXt models. As shown in the third column of Figure 11 which shows a case of papillary muscle interference, there is notable incorrect segmentation for the U-Net, UNet++, and UNeXt models. For these cases, the Attention U-Net model performs much better than the U-Net, UNet++, and UNeXt models, with the left ventricle segmentation quite close to the ground truth. The reason may be that the attention mechanism incorporated in the Attention U-Net model could well deal with the challenging issues of left ventricle boundary missing and papillary muscle interference. Further research is required to resolve the problems.

Based on the promising results of the current study, it is strongly anticipated that further research into real-time assessment of left ventricle function and structure will proceed smoothly.

## 5. Conclusions

The current study highlights the feasibility of using deep learning for left ventricle segmentation from TSV on TEE, with promising accuracy and speed, based on a large TEE dataset. The performances of U-Net and its variants are comparable. It potentially facilitates an accelerated and objective alternative for cardiovascular assessment in perioperative management. Further research is required to explore its application in challenging cases and real-time assessment of left ventricle function and structure.

## Figures and Tables

**Table 1:** Left ventricle segmentation in TSV TEE images with respect to the number of model parameters, training time, and inference time for a single image of the U-Net, UNet++, Attention U-Net, and UNeXt models. TEE: transesophageal echocardiography; TSV: transgastric short-axis view.

Deep Learning Models	# of Model Parameters	Training Time	Inference Time for a Single Image
U-Net [18]	7.85 million	6428.65 s	101.75 ms
UNet++ [19]	9.16 million	10,080.50 s	134.21 ms
UNeXt [20]	1.47 million	7122.94 s	109.59 ms
Attention U-Net [21]	34.88 million	10,556.86 s	122.85 ms

**Table 2:** Left ventricle segmentation performance of U-Net, UNet++, Attention U-Net, and UNeXt on the test set of TSV TEE images (*n* = 138) evaluated using JSC and DSC. Data are expressed as mean ± standard deviation. TEE: transesophageal echocardiography; TSV: transgastric short-axis view.

Deep Learning Models	JSC (%)	DSC (%)
U-Net [18]	84.71 ± 10.25	90.98 ± 7.19
UNet++ [19]	86.02 ± 8.70	91.76 ± 5.48
UNeXt [20]	84.20 ± 9.62	91.00 ± 6.23
Attention U-Net [21]	85.93 ± 8.71	92.00 ± 5.50

## Data Availability

Data is unavailable due to ethical restrictions.
